# Lipedema: Clinical Features, Diagnosis, and Management

**DOI:** 10.1055/a-2530-5875

**Published:** 2025-05-15

**Authors:** Hatan Mortada, Abdulmalek W. Alhithlool, Nouf Z. AlBattal, Rashika K. Shetty, Ghaleb A. AL-Mekhlafi, Joon Pio Hong, Feras Alshomer

**Affiliations:** 1 Division of Plastic Surgery, Department of Surgery, King Saud University Medical City, King Saud University and Department of Plastic Surgery & Burn Unit, King Saud Medical City, Riyadh, Saudi Arabia; 2 College of Medicine, King Faisal University, Alhasa, Saudi Arabia; 3 College of Medicine, King Saud bin Abdulaziz University for Health Sciences, Riyadh, Saudi Arabia; 4 University of Minnesota Medical School, Minneapolis, Minnesota; 5 College of Medicine, Fakeeh College for Medical Sciences, Jeddah, Saudi Arabia; 6 Department of Plastic and Reconstructive Surgery, University of Ulsan College of Medicine, Asan Medical Center, Seoul, Korea.; 7 Division of Plastic Surgery, Department of Surgery, King Saud bin Abdulaziz University for Health and Sciences, Riyadh, Saudi Arabia

**Keywords:** lipedema, lymphedema, liposuction

## Abstract

Lipedema is an adipose tissue disorder that principally affects women and is frequently misidentified as obesity or lymphedema. There have been relatively few studies that have precisely defined the pathogenesis, epidemiology, and treatment approaches for lipedema. However, successfully recognizing lipedema as a distinct condition is important for proper management. This review aimed to examine the existing literature on the epidemiology, pathogenesis, clinical presentation, differential diagnosis, and treatments for lipedema. The current research indicates that lipedema appears to be a clinical entity related to genetic factors and fat distribution, although distinct from lymphedema and obesity. Some available treatments include complex decongestive physiotherapy, liposuction, and laser-assisted lipolysis. The management of lipedema is complex and differs from that of lymphedema. Further high-quality randomized controlled trials are urgently needed to continue advancing our understanding of this often neglected disease and exploring optimal medical and surgical treatment regimens tailored specifically for lipedema patients. In summary, despite frequent misdiagnosis, enhanced recognition, and research into customized therapeutic strategies for this poorly characterized but likely underdiagnosed disorder represent promising steps forward.

**Level of evidence** N/A.

## Introduction

Lipedema is a condition affecting adipose tissues that was first identified by Wold et al. 1 The disease is characterized by its progressive nature, attributed to the abnormal accumulation of subcutaneous fat. This leads to developing bilateral lower limb and/or upper extremity enlargement and swelling. Moreover, lipedema typically spares the hands, feet, and trunk; however, the arms are frequently affected. 2 3 4 It is crucial to note that the actual cause of the disease is yet unknown. 5

Primarily, females between puberty and the third decade of life, are more susceptible to and predominantly affected by this condition than males. 1 6 Moreover, stressful life events, trauma, or surgical procedures can trigger the onset of lipedema. 4 Notably, there is a belief in the potential inheritance of lipedema, which may occur through either autosomal dominant or X-linked patterns with sex limitation. Although the specific genes and proteins associated with lipedema are not fully understood and detectable biomarkers in lipedema patients remain limited, genetic inheritance has been observed in approximately 60% of cases, with suggestions of the involvement of multiple genes. 3 7 8 Furthermore, lipedema can contribute to various health complications, including hypothyroidism, electrolyte imbalance due to the disposition of sodium within the skin, cardiovascular problems, and reduced levels of vitamin D, particularly in cases where the body mass index (BMI) surpasses average values. Additionally, psychological distress may ensue, leading to a compromised quality of life among affected individuals. 9 10 11 12 13 14 15 16 17

Lipedema is a clinical diagnosis requiring extensive knowledge and identification of the condition, proper history taking, and physical examination. 18 However, it is possible to misdiagnose it due to the circumferential enlargement of the legs, which is comparable to lymphedema and nonlipedema obesity. Apart from this, lipedema can be distinguished after multiple attempts of weight reduction that have shown to have modest effects on the lower extremities. 8 19 20 21 Long-term lipedema may result in secondary lymphedema due to mechanical pressure on the lymphatic system due to fatty hypertrophy. Additionally, elevated sodium levels may lead to the recruitment of inflammatory mediators that directly impede lymphatic evacuation, exacerbating edema production, especially in the latter stages where lymphedema frequently coexists with lipedema. 22 23 24 25 26 As a result, diagnosis might be delayed for up to 10 years. 27

Hence, there is an urgent need to comprehensively review the existing literature on lipedema to delineate its distinct epidemiology, pathogenesis, diagnostic features, and tailored therapeutic approaches. This review aims to provide clearer insight into this perplexing condition and inform future research directions by evaluating the current knowledge gap and limitations of prior studies. The goal is to promote early, accurate diagnosis and appropriate multimodal care of lipedema patients through evidence-based recommendations. Enhanced understanding of this distinct disorder is imperative to develop more effective therapies and improve prognosis and life quality for those affected.

## Methods and Materials

Articles for this narrative review, conducted in January 2024, were gathered from the Google Scholar and PubMed databases. The search terms “Lipedema,” “Lipoedema,” “Lipoedemas,” and “Lipolymphedema” were employed. After thorough screening and searching, thirty publications were selected to address the research objectives. 15 20 26 28 29 30 31 32 33 34 35 36 37 38 39 40 41 42 43 44 45 46 47 48 49 50 51 52 53 54 The inclusion criteria for this narrative review are as follows: studies focusing on lipedema in human subjects were selected. Both research articles and reviews published in peer-reviewed journals were included to ensure the credibility and reliability of the sources. The studies needed to provide clinical insights into lipedema's characteristics, diagnosis, or management. Only publications available in English were considered to ensure accessibility for review and analysis. The review encompassed studies across different age groups, including both pediatric and adult populations. Additionally, research exploring diverse management strategies for lipedema was included to provide a comprehensive understanding of the condition. The assessment involved reviewing relevant article titles and abstracts, with subsequent retrieval of full text.

## Narrative Review

### Epidemiology and Prevalence of Lipedema

Worldwide, very few papers report the prevalence of lipedema, as it is a frequently misdiagnosed or underdiagnosed disease. It is estimated that the prevalence of lipedema is around 11%, ranging between 15 and 18% in the United States and across Europe, respectively. 18 29 33 34 These prevalence estimates are likely underestimates due to the high frequency of misdiagnosis and underdiagnosis of lipedema.

### Demographic Characteristics

Although primarily known as an exclusively female disease, lipedema has rarely been reported in men. Onset often occurs during puberty, the third decade of life, or after events like menopause and childbirth, suggesting a link to hormonal fluctuations like estrogen. 3 15 Lipedema is associated with high BMI for both overweight and obesity. 15 Despite normal upper body habitus, lipedema patients exhibit disproportion between the arms and legs, with a waist-to-hip ratio <1. 3 6 Lipedema showed potential inheritance, from 16 to 64% of patients report a family history of lipedema. 15 55 A study in Brazil correlated anxiety, depression, hypertension, and anemia with increased lipedema risk in women. 46

### Pathophysiology

To date, no pathophysiological mechanism has been identified; however, several theories have been proposed, mainly revolving around the hormonal influence of estrogen, abnormal adipogenesis, and pathological angiogenesis. Estrogen plays a significant role in the body, especially in females. 31 One is controlling genes that lead to endothelial barrier disruption, as Szel et al stated. Additionally, it is involved in the sympathetic innervation of fatty tissue, explaining the neuropathy associated with lipedema. 2 29 Lipedema is characterized by its increased palpation sensitivity, attributed to adipocyte necrosis and macrophages secondary to hypoxia from increased fat deposition. 29 33 56 57 Another theory suggests abnormal angiogenesis and loss of elasticity. Although lymphatic vessels and capillaries typically lack elastic tissue, the surrounding connective tissue possesses elasticity. 33 Therefore, in such cases, lymphatic vessels lose their ability to open properly under increased pressure within the extracellular matrix, resulting in leakage from the capillaries. 15 58 Moreover, Siems et al propose that hypoxic adipose tissue induces the proliferation of blood vessels by overly stimulating vascular endothelial growth factors. 15 59

### Genetic Factors

Lipedema is inherited via X-linked dominant or autosomal dominant patterns with a gender bias toward females. This condition can be divided into two main types: syndromic and nonsyndromic. 31 In syndromic lipedema, notable genetic mutations include: the PIT-1 mutation, affecting anterior pituitary gland hormone secretion—specifically growth hormone, prolactin, and thyroid-stimulating hormone—leading to potential hormone deficiencies and lipedema. 31 33 60 61 62 Another genetic condition, Sotos Syndrome, features mutations or microdeletions in the NSD1 gene and is characterized by early excessive growth, macrocephaly, and developmental delays, with some diagnosed cases also presenting lipedema. 31 60 63 Lastly, Williams Syndrome, caused by a microdeletion in chromosome 7 that results in elastin gene loss, has been observed with a unique lipedema phenotype, including reduced bone mass and a predominance in males without pain or tenderness sensitivity. 31 33 64

For nonsyndromic lipedema, mutations in the aldo-keto reductase gene have been implicated. This gene plays a crucial role in converting active progesterone into its inactive form. A mutation here can disrupt this process, resulting in progesterone accumulation that may contribute to abnormal fat deposition, a hallmark of lipedema, and associated weight gain. 31 65

### Clinical Manifestation and Diagnosis

**Fig. 1:**
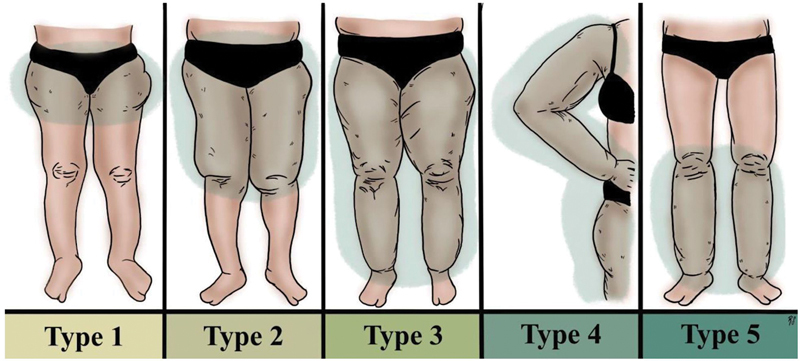
Lipedema types.

**Fig. 2:**
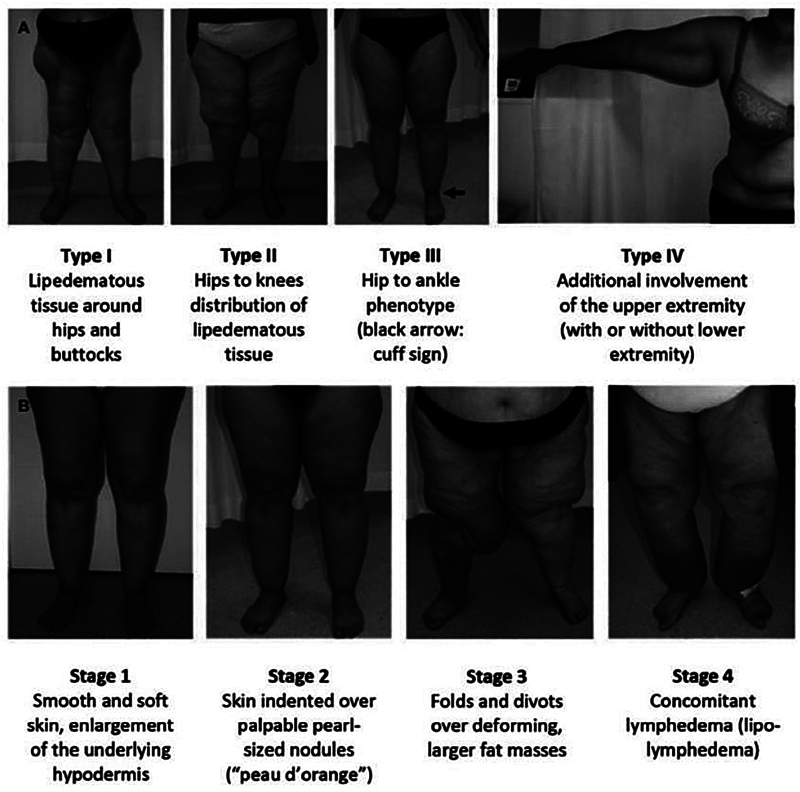
This figure illustrates the ( **A** ) various forms and ( **B** ) progression phases of lipedema. This figure is reproduced with permission from Bouillon et al. 44

Lipedema primarily presents as an enlargement of the lower limbs, and may also symmetrically affect the upper limbs. It is characterized by clear demarcations at the feet and hands, leading to the distinctive “cuff sign.” The distribution of fat is disproportionately concentrated on the outer regions of the buttocks, thighs, and calves while sparing the abdomen, feet, and hands. The condition is classified into five types based on the pattern of fat distribution, as detailed in Table 1 and depicted in Fig. 1 . 15 Among these types, the most commonly observed are types 1 to 3, compared with types 4 and 5 ( Fig. 1 ). As for the severity, Herbes et al have classified lipedema into stage 1, involving an enlarged hypodermis with smooth and intact skin; stage 2, characterized by the nodular changes in adipose tissue with irregular skin surface; stage 3, characterized by contour deformity of the knees and thighs as a result of the considerable growth of nodular fat, and stage 4 involving the coexistence of lymphedema ( Fig. 2 ). 31 34 66 67 68 69 70 In their comprehensive guidelines for the diagnosis of lipedema, Halk and Damstra present a detailed framework, as outlined in Table 2 , which enumerates critical diagnostic criteria and considerations for clinicians and researchers. 66 Key features distinguishing lipedema from lymphedema and obesity include susceptibility to bruising, pain upon palpation, and progressive enlargement that does not respond to lifestyle changes. The pain associated with lipedema is often described as a pressure-like, dull, and heavy sensation, primarily affecting the anterior tibial and femoral regions. This discomfort may be provoked by light touch and exacerbated by prolonged standing or sitting, becoming particularly noticeable toward the day's end. 30 40 54 Joint hypermobility is another characteristic, with studies indicating its prevalence between 44 and 58% among lipedema patients. 11 34 Additionally, the condition may manifest with telangiectasia, giving the skin a threadlike appearance of dilated blood vessels, and cold extremities. 34 Table 3 details the clinical characteristics of lipedema patients, highlighting key symptoms and diagnostic features. Obesity is a prevalent comorbidity in lipedema, with its progression closely tied to the worsening of lipedema symptoms. Bertsch and Erbacher found that 88% of their patients had obesity (BMI > 30 kg/m ^2^ ), suggesting that patients of average weight are rare. However, contrasting findings indicate that 40% of patients had a normal BMI, yet exhibited significant disproportion between their upper and lower bodies. 34 71

**Table 1:** Classification of lipedema by type and affected areas

Type	Description
I	Increased fat deposits in the thighs, hips, and glutes
II	Involves the knees with a fat pad in the internal zone
III	Reaches down to the ankles
IV	Involves the upper limbs
V	Involves only the lower legs

**Table 2:** Halk and Damstra guidelines for lipedema diagnosis 66

A) Criteria of Wold et al 1 1) Bilateral and symmetrical involvement of lower limbs, except the feet 2) Nonpitting edema 3) Painful, tender, and easily bruised 4) Unresponsive to diet and weight reduction interventions 5) Unresponsive to diet and weight reduction interventions
B) Physical exam Proximal lower limb 1) Disproportionate fat arrangement with the body above the waist (“riding breeches”) 2) Thick cutaneous fat in a circumferential fashion Distal lower limb 1) Proximal subcutaneous fat thickness 2) Distal subcutaneous fat thickness, sparing the feet, creating a sharp demarcation known as the “cuff sign” Proximal upper limb 1) Significant fat accumulation (“angel wing sing”) 2) Abrupt stop at the elbows Distal upper limb 1) Distal subcutaneous fat thickness, sparing the hands (“handcuff sign”)
C) Extra criteria 1) Pain with bimanual palpation 2) Distal fat tissue tendrils of the knee

**Table 3:** Clinical characteristics of lipedema patients

Characteristic	
A.	Bilateral lower extremity involvement
B.	Progression to lipo-lymphedema
C.	Early bilateral involvement, late arm involvement (>30% of cases)
D.	Fat accumulation in limbs vs. uniform in obesity
E.	Adipose resistance to diet and exercise
F.	Tenderness and pain upon palpation (painful fat syndrome)

Diagnosing lipedema is challenging, often leading to misdiagnosis. To address this, diagnostic criteria were established to enhance clinical suspicion. Initially, Wold et al introduced key determinants for narrowing down differentials. 1 Subsequently, the Dutch work group developed comprehensive guidelines that include the Wold et al criteria 1 physical examination findings, and additional criteria, detailed in Table 1 . A diagnosis of lipedema is highly probable when all five criteria from Wold et al and two physical examination findings are present. If these criteria are not fully met, the presence of any two additional criteria may still support a diagnosis.

### Differential Diagnoses

Several conditions are frequently confused with lipedema, which are lymphedema, obesity, and venous insufficiency; therefore, the distinction between them is crucial to reaching the correct diagnosis. 32 40

#### Lymphedema

Lymphedema involves lymphatic drainage dysfunction leading to limb swelling from lymph accumulation, whereas lipedema stems from localized adipose tissue deposition. 15 40 Clinically, lymphedema initiates distally and unilaterally with a possible Stemmer's sign, which, when positive, means the inability to pinch and lift a fold of skin at the base of the second toe or finger. Lipedema is bilateral, spares feet, causes pain/bruising, and lacks Stemmer's sign. 15 40 Table 4 provides an overview of the clinical disparities observed between the two conditions. Imaging aids diagnosis. Ultrasound shows increased subcutaneous thickness and decreased echogenicity in lymphedema, whereas lipedema has normal thickness and echogenicity. 33 40 MRI offers high sensitivity in differentiating the conditions. Both MRI and CT reveal homogenous subcutaneous fat thickening in lipedema versus fluid accumulation and honeycombing with muscle hypertrophy in lymphedema. 32 40 Other modalities, such as indocyanine green lymphography (ICG) lymphography and scintigraphy, show normal or increased lymphatic function in lipedema. Additionally, the tissue dielectric constant and DEXA can provide further distinction if ambiguity remains. 32 40

**Table 4:** Clinical differences between lipedema and lymphedema

	Lipedema	Lymphedema
Gender predominance	Female	Both male and female, equally
Affected limbs	Symmetric and bilateral	Asymmetric, could be uni- or bilateral
Hands/feet involvement	Spared	Involved
Skin color	Brown/warty/sclerotic	Intact skin
Stemmer sign	Negative	Positive
Tenderness	Yes	No
Pitting edema	Absent	Yes (unless chronic)
Consistency	Soft	Firm/tense
Bruising	Present	Not present
Compression therapy	Unresponsive	Responsive
Weight reduction therapy	Unresponsive	Responsive

ICG offers a pivotal diagnostic approach for lymphoedema by employing a medical dye to map lymphatic flow, highlighting the efficiency or dysfunction within the system. This method visualizes lymphatic transport, distinguishing between normal and pathological conditions, such as dermal backflow in lymphoedema cases. ICG Lymphography's ability to assess lymphatic functionality in real-time is crucial for developing targeted treatment plans. 72

#### Obesity and Localized Areas of Fat Deposition

Differentiating lipedema from obesity is challenging as it can co-occur. However, the key distinction is that obesity improves with lifestyle changes like diet and exercise, whereas lipedema does not. Also, obesity has a more proportional fat distribution compared with lipedema's isolated limb involvement. 15 32 40 Localized adiposity in obesity can resemble lipedema but is more responsive to interventions. Dercum's disease and Madelung's diseases are rare adipose disorders with distinct features, such as multiple lipomas or male predisposition. 32 40 Lipedema's poor responsiveness to weight loss and disproportionate fat distribution help distinguish it from generalized or localized obesity.

#### Venous Insufficiency

Chronic venous disease (CVD) is another condition confused with lipedema. 32 However, CVD has distinct features like skin discoloration, varicose veins, pitting edema, and ulceration that improve with interventions like elevation, compression, and exercise. Lipedema lacks these hallmarks. Though varicose veins occur in both conditions, a history of ulceration or cellulitis makes CVD more likely. Additionally, cellulitis is frequently associated with lymphatic dysfunction. 32 40 Overall, the amenability of CVD signs to treatment versus lipedema's resistance helps differentiate the two.

### Psychosocial Impact

Lipedema profoundly impacts quality of life, including physical, emotional, and social functioning. 8 29 41 55 73 Clarke et al found that patients with advanced lipedema (stages 3 and 4) reported significantly higher rates of mobility issues, pain, fatigue, and work problems compared with those with early-stage lipedema (stages 1 and 2). Advanced-stage patients also experienced more mental health issues, including depression and feelings of isolation. 41 Misdiagnosis is common, with 41% diagnosed after age 40. 41 48 Lipedema dismissal as an “invalid condition” was reported by 30%. 41 Due to misperception of obesity, lifestyle modifications are often advised, leading to self-doubt when ineffective. One study found that patients felt that doctors' advice on diet/exercise reflected ignorance, further postponing diagnosis/treatment. 49 Healthcare weight stigma and misinformation must be addressed given their detrimental impacts on care and psychological outcomes. 41 74 Disease progression impairs mobility and independence due to accumulating joint deterioration, pain, and fatigue. 9 39 40 41 A study of stages 3 and 4 patients revealed significant mobility/activity limitations affecting work. 41 75 More support may be needed in the late stages to maintain functioning. Psychological distress varies by stage. Failed weight loss efforts in adolescence often trigger long-term cycles of eating disorders in up to 18% of individuals. 41 54 76 77 Depression prevalence ranges from 31 to 59%, 41 54 76 77 higher with a BMI ≥ 40. 77 Perceived unmanaged symptoms like pain also worsen anxiety and depression. 59 Despite profound impacts, the current quality of life is considered moderate. 39

### Treatment Approaches

Lipedema's complex, poorly understood pathophysiology, and limited therapeutic options pose significant clinical challenges. Patient education is crucial to align expectations with available management approaches, which aim to relieve symptoms, improve functioning, slow progression, and emphasize secondary prevention. 32 45 Various specialties, including plastic surgery, vascular surgery, and rehabilitation, diagnose and treat lipedema across countries. 70 The inability to cure lipedema represents a major global challenge requiring further research. 70 A multimodal approach encompassing psychosocial support, education, and family planning. 34 The mainstays of treatment include conservative measures and surgical procedures. 40 Tables 5 and 6 outline the classification, assessment, and treatment options for lipedema, providing a structured approach to management strategies.

**Table 5:** Classification and treatment of lipedema

Category	Type/approach	Description
Phenotypes		
	Columnar	Enlargement of lower extremity portions through conic sections
	Lobar	Large bulges or lobes in the hip region or lower extremities
Types I–V lipedema		
	Type I	Pelvis, buttocks, hips
	Type II	Buttocks to the knees with specific fat pads and fold around the knee
	Type III	Buttocks to the ankles
	Type IV	Arms
	Type V	Isolated lower leg
Herbst classification		
	Stage I	Intact skin surface with enlarged hypodermis
	Stage II	Nodular shape changes in subcutaneous fat with uneven skin
	Stage III	Huge nodular fat growths around thighs and knee side causing contour deformity
	Stage IV	Advanced lipedema, with lipo-lymphedema
Treatment of lipedema		
Conservative treatment		
	Complex decongestive therapy	Manual lymph drainage (MLD), skincare, multilayer compression bandaging, exercise
	Compression garments	
	Pneumatic compression	
	Exercise	
Surgical treatment		
	Debulking surgeries	
	Lipoaspiration	
	Tumescent lipoaspiration	

**Table 6:** Assessment and diagnosis of lipedema

History taking	Onset of duration and symptoms
	Progression/exacerbation of symptoms
	Previous treatment modalities
	Estrogen disorders or imbalances
Physical examination	
	Extent of swelling
	Asymmetry/symmetry
	Surgical scars or other skin conditions
	Fat pads, signs of chronic venous insufficiency
	Godet's sign (pitting edema)
	Stemmer's sign (lymphedema)
Investigations	
	Magnetic resonance imaging (MRI)
	Ultrasound
	Tissue dielectric constant
Diagnosis criteria	
	Gender predisposition (female)
	Overweight
	Painful limbs
	Diet-resistant weight
	Easy bruising
	Negative Stemmer's sign
	Sparing of the feet
	Nonpitting edema
	Symmetrical enlargement

### Conservative Strategies

Standard conservative therapy for lipedema includes dietary counseling, manual therapy, compression garments, pneumatic compression devices, and home exercise plans. 20

#### Decongestive Therapy

Complex decongestive therapy (CDT) is used for both lipedema and lymphedema but shows limited benefit in pure lipedema without fluid excess or lymphatic dysfunction. 34 Guidelines propose CDT as a first-line conservative treatment, 30 though its components—exercise, compression bandaging, manual lymph drainage, and skincare—only reduce limb circumference by up to 10%. 30 Pneumatic compression augments CDT by improving venous return and can be used at home when combined with patient education. 78 Overall, CDT may offer mild improvements in lipedema but is likely more effective for concurrent lymphedema.

#### Compression Therapy

Compression garments streamline limb shape and reduce lipedema symptoms like heaviness, pain, and edema. 69 70 Decongestive bandaging should precede compression in active edema. Compression does not reduce fat but can prevent new edema while improving lymphatic/venous flow to alleviate associated problems. 32 Daily compression use is critical for maintaining CDT benefits and improving symptoms and function. However, only 38% of patients comply, likely due to skin sensitivity. Compression provides symptomatic relief but requires careful introduction and consistent use to optimize efficacy. 78 79

#### Massage

Subcutaneous adipose tissue therapy, known as quadrivas therapy, utilizes deep scraping and hook massage techniques to stimulate microcirculation. The therapy has significantly reduced weight, leg fat mass, and total leg volume in patients. 30

#### Physical Activity

Physical activity in lipedema should aim to achieve realistic weight loss and control symptoms. Exercise preferentially affects the arms versus legs, 69 80 but increasing leg muscle activity can improve lymphatic drainage and prevent edema—additional well-being, self-esteem, mobility, and strength. 34

#### Dietary Program

Dietary changes alone cannot reduce lipedema fat accumulation but may improve prognosis and well-being when combined with obesity management. 81 82 83 Early weight loss and diet modifications could lessen inflammation, preventing symptom deterioration. 57 Diet should be combined with lifestyle changes like exercise, walking, swimming, and compression. 29 40 Overall, while unable to directly treat lipedema, dietary interventions play a supportive role in managing secondary obesity and inflammation. While no specific diet is approved for lipedema, nutrition remains an important component of treatment. 40 Some evidence suggests ketogenic diets may aid lipolysis. 39 One case study described substantial weight loss (−41 kg) and reduced limb circumference in a patient on a long-term ketogenic diet, who also reported improved pain and quality of life after declining other treatments. 42 This example illustrates the potential of customized ketogenic diet plans for lipedema management. The Mediterranean diet, abundant in fruits and vegetables and characterized by a reduced carbohydrate intake of around 40% of total calories, has shown partial effectiveness in managing lipedema. This benefit is likely due to better blood sugar control from reduced carbohydrates and increased fiber. Moreover, the diet's rich content of vitamins and polyphenols, which have recognized epigenetic effects, contributes to its health benefits. These factors help reduce inflammation and improve metabolic health, potentially alleviating some symptoms of lipedema. 42 Overall, nutrition is adjunctive, and individualized dietary plans like ketogenic diets warrant further research.

### Surgical Interventions for lipedema

**Fig. 3:**
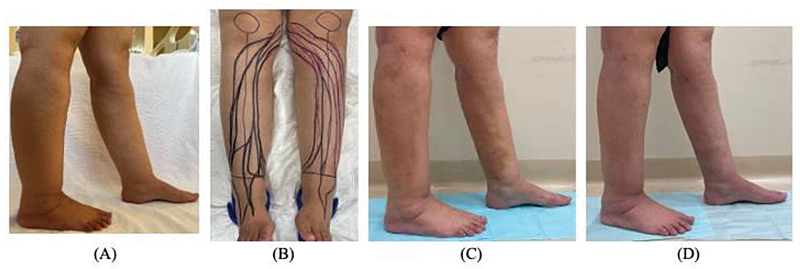
A patient was diagnosed with lipedema. ( **A** ) Preoperative condition. ( **B** ) Intraoperative indocyanine green lymphography. ( **C** ) 3 weeks postoperative. ( **D** ) 2 months postoperative, showing marked improvement.

Conservative management should precede surgical options. 33 Surgery is the only treatment that reduces lipedema fat, decreases volume and prevents mechanical impairment. 40 The two main surgeries are liposuction and lipectomy, with liposuction being the most common. 32 However, surgery, especially liposuction, is costly. 33 Some advise against surgery due to risks of subsequent lymphedema ( Fig. 3 ). 57 84

#### Liposuction

Multiple liposuction techniques have been developed, each offering distinct advantages. Initially, dry liposuction, prevalent before the 1990s and performed under general anesthesia, was associated with lymphatic damage, edema, and bleeding. In contrast, wet liposuction techniques, including tumescent, super wet, and wet liposuction, involve the infiltration of fluid before aspiration. This approach helps preserve the lymphatics and reduces complications, making wet liposuction, which uses a combination of vasoconstrictor and anesthetic in saline, safer and less traumatic compared with dry liposuction. 34 85 Among these, tumescent liposuction is regarded as the gold standard, efficiently removing fat with minimal impact on lymphatics. 15 It is subdivided into water-jet-assisted and vibration-assisted techniques, with the latter being more prevalent and further minimizing tissue injury. 86 87 For younger, nonobese patients, laser-assisted liposuction offers superior outcomes in terms of skin tightening and limb contouring. However, its effectiveness is less pronounced in elderly patients with lipedema. 30 Mega-liposuction, which requires careful monitoring of fluid and electrolytes, presents another option. 34 Undergoing multiple liposuction sessions may also contribute to an improved overall health status. 44 A study involving 85 lipedema patients demonstrated the long-term effectiveness of liposuction through a questionnaire evaluation. 40 To address lymphatic damage, specific techniques such as tumescent liposuction and the use of small cannulae are employed for treating lipedema. 53 A recent study by Mortada et al assessed various liposuction modalities in managing lipedema and found that only a small percentage of patients (0.18%) developed secondary lymphedema following the procedure. 88 Yamamoto et al demonstrated the use of ICG lymphography to visualize and preserve main lymph flows during leg lipectomy procedures, potentially reducing postoperative complications such as seroma and wound dehiscence. 52 Additionally, mapping the lymphatics using ICG lymphography during surgery helps visualize and thus minimize lymphatic damage, aiming to reduce the risk of secondary lymphedema. 52 89 90 Given the potential complications, these procedures should ideally be performed by experienced professionals. After the removal of excess fat from the body, patients have reported decreased pain, muscle contractions, tightness, itching, swelling, and bruising, as well as improved physical appearance. Surgical management decreases bulkiness and enhances ambulation biomechanics. In addition, a few studies have shown an increase in life satisfaction, including reduced ache, fullness, and weariness, along with improvements in physical health conditions and mental well-being in general. Nevertheless, it is worth noting that liposuction is very costly. 28 36

#### Lipectomy

Lipectomy is a more invasive debulking procedure involving the excision of substantial localized fat deposits. It is reserved for advanced lipedema with severe mechanical impairment or limb abnormalities. 34 Factors like patient age, disease duration, and length of conservative therapy do not appear to significantly impact surgical outcomes. 32

### Emerging Approaches: Pharmacotherapy and Devices

While no single pharmacological agent is approved for lipedema, drugs can address associated symptoms and complications. Goals include minimizing pain, swelling, fibrosis, inflammation, and preventing venous disease and secondary lymphedema. 34 47 Metformin and resveratrol may prevent fibrosis and inflammation. Metformin benefits those with metabolic abnormalities. Sympathomimetics like amphetamine promote lipolysis through adrenoreceptors on vessels. 8 18 91 Diosmin reduces swelling from chronic venous insufficiency. Selenium improves inflammation and leg volume. 8 18 91 Drugs causing fluid retention should be avoided. Furosemide increases interstitial proteins, impeding fluid removal. 20 33 When appropriate, home pneumatic compression can better control swelling, pain, and lymph flow versus self-manual drainage, and reduces postoperative DVT risk when combined with early mobilization. 92 93 94

### Future Directions

Several critical knowledge gaps remain in lipedema pathophysiology. While chronic inflammation is a known hallmark, the specific inflammatory pathways driving adipose expansion, immune responses, and angiogenesis require further elucidation to identify therapeutic targets. 30 45 The mechanisms underlying the predominant lower extremity adipose distribution are unclear and warrant investigation. 45 The factors driving lipedema progression from early to advanced stages are unclear and require study to enable targeted interventions. 41 Risk factors like genetics and environment are uncertain despite suspected heritability. 48 Altered estrogen receptor content implies sex hormone involvement but needs further research. 51 Comorbidities with lymphedema and obesity add complexity. 15 30 33 Bridging knowledge gaps in lipedema pathogenesis will enable the evolution of more effective future management strategies and enhanced quality of life for those affected.

Further investigation into the pathogenesis of lipedema is crucial, focusing on inflammatory pathways, molecular mechanisms driving adipose deposition, determinants of disease progression, and identification of risk factors. Improving diagnosis and awareness is paramount for patients with this condition's well-being. This will require establishing standardized diagnostic criteria, epidemiological studies, and initiatives to educate healthcare professionals. Additionally, optimizing treatment strategies and patient support should involve personalized approaches, longitudinal studies on surgical interventions, and integration of psychosocial support into patient care. Multidisciplinary approaches and a holistic understanding of lipedema are vital for improving early recognition, access to care, and overall quality of life for individuals affected by this complex condition. By addressing these recommendations, future research can contribute to developing more effective strategies for managing and treating lipedema, ultimately enhancing the well-being of those impacted by this challenging disorder.

## Conclusion

According to the studies included in this review, lipedema is a frequently misdiagnosed and underdiagnosed disease, characterized by disproportionate fat accumulation, primarily affecting the lower limbs, and having a far greater prevalence in women. The condition is associated with hormonal influences and genetic and demographic factors, though further research is needed to understand its pathogenesis and interplay with lymphedema and obesity. Treatment approaches for lipedema include surgical interventions, conservative strategies, emerging therapies, and multidisciplinary care.

## References

[1] Wold L E Hines E A Jr Allen E V Lipedema of the legs; a syndrome characterized by fat legs and edema Ann Intern Med 1951 34 05 1243 1250 14830102 10.7326/0003-4819-34-5-1243

[2] Szél E Kemény L Groma G Szolnoky G Pathophysiological dilemmas of lipedema Med Hypotheses 2014 83 05 599 606 25200646 10.1016/j.mehy.2014.08.011

[3] Child A H Gordon K D Sharpe P Lipedema: an inherited condition Am J Med Genet A 2010 152A 04 970 976 20358611 10.1002/ajmg.a.33313

[4] Buck D W II Herbst K L Lipedema: a relatively common disease with extremely common misconceptions Plast Reconstr Surg Glob Open 2016 4 09 e1043 27757353 10.1097/GOX.0000000000001043 PMC5055019

[5] van la Parra R FD Deconinck C Pirson G Servaes M Fosseprez P Lipedema: what we don't know J Plast Reconstr Aesthet Surg 2023 84 302 312 37390539 10.1016/j.bjps.2023.05.056

[6] Fife C E Maus E A Carter M J Lipedema: a frequently misdiagnosed and misunderstood fatty deposition syndrome Adv Skin Wound Care 2010 23 02 81 92, quiz 93–94 20087075 10.1097/01.ASW.0000363503.92360.91

[7] Amato A C Amato L L Benitti D Amato J L Assessing the Prevalence of HLA-DQ2 and HLA-DQ8 in lipedema patients and the potential benefits of a gluten-free diet Cureus 2023 15 07 e41594 37431427 10.7759/cureus.41594 PMC10329849

[8] Herbst K L Rare adipose disorders (RADs) masquerading as obesity Acta Pharmacol Sin 2012 33 02 155 172 22301856 10.1038/aps.2011.153 PMC4010336

[9] Herbst K L Mirkovskaya L Bharhagava A Chava Y Hanne C Te T Lipedema Fat and Signs and Symptoms of Illness, Increase with Advancing StageAvailable at:http://wwwimedpub.com(Accessed on December 19, 2023)

[10] Bauer A T von Lukowicz D Lossagk K New Insights on lipedema: the enigmatic disease of the peripheral fat Plast Reconstr Surg 2019 144 06 1475 1484 31764671 10.1097/PRS.0000000000006280

[11] Beltran K Herbst K L Differentiating lipedema and Dercum's disease Int J Obes (Lond) 2017 41 02 240 245 27857136 10.1038/ijo.2016.205

[12] Pereira-Santos M Costa P RF Assis A MO Santos C AST Santos D B Obesity and vitamin D deficiency: a systematic review and meta-analysis Obes Rev 2015 16 04 341 349 25688659 10.1111/obr.12239

[13] Schmeller W Schmeller W Meier-Vollrath I Lipödem: ein update LymphForsch. 2005 9 01 10 20

[14] Forner-Cordero I Szolnoky G Forner-Cordero A Kemény L Lipedema: an overview of its clinical manifestations, diagnosis and treatment of the disproportional fatty deposition syndrome - systematic review Clin Obes 2012 2(3-4):86 95 25586162 10.1111/j.1758-8111.2012.00045.x

[15] Okhovat J P Alavi A Lipedema: a review of the literature Int J Low Extrem Wounds 2015 14 03 262 267 25326446 10.1177/1534734614554284

[16] Nemes A Kormányos Á Domsik P Kalapos A Kemény L Szolnoky G The impact of lower body compression garment on left ventricular rotational mechanics in patients with lipedema-Insights from the three-dimensional speckle tracking echocardiographic MAGYAR-Path Study Clin Obes 2020 10 05 e12380 32573965 10.1111/cob.12380

[17] Crescenzi R Marton A Donahue P MC Tissue sodium content is elevated in the skin and subcutaneous adipose tissue in women with lipedema Obesity (Silver Spring) 2018 26 02 310 317 29280322 10.1002/oby.22090 PMC5783748

[18] Herbst K L Subcutaneous adipose tissue diseases: dercum disease, lipedema, familial multiple lipomatosis, and madelung disease. Endotext. Accessed 2019 at:https://www.ncbi.nlm.nih.gov/books/nbk552156/

[19] Buso G Depairon M Tomson D Raffoul W Vettor R Mazzolai L Lipedema: a call to action! Obesity (Silver Spring) 2019 27 10 1567 1576 31544340 10.1002/oby.22597 PMC6790573

[20] Herbst K L Kahn L A Iker E Standard of care for lipedema in the United States Phlebology 2021 36 10 779 796 34049453 10.1177/02683555211015887 PMC8652358

[21] Fonder M A Loveless J W Lazarus G S Lipedema, a frequently unrecognized problem J Am Acad Dermatol 2007 57 02 S1 S3 17637360 10.1016/j.jaad.2006.09.023

[22] Bilancini S Lucchi M Tucci S Eleuteri P Functional lymphatic alterations in patients suffering from lipedema Angiology 1995 46 04 333 339 7726454 10.1177/000331979504600408

[23] Jagtman B A Kuiper J P Brakkee A JM Wijn P FF Mesures de l'elasticite de la peau chez des personnes normales et chez des malafes souffrant d'une insuffisance veineuse chronique Phlébologie (Paris) 1983 36 01 57 65 6836022

[24] Földi E Földi M Tischendorf F Adipositas, lipedema and lymphostasis Med Welt 1983 34 07 198 200 6835084

[25] Microlymphatic aneurysms in patients with lipedema. Accessed Feb 23, 2024 at:https://www.researchgate.net/publication/11573924_microlymphatic_aneurysms_in_patients_with_lipedema 11783595

[26] Tarbell J M Simon S I Curry F RE Mechanosensing at the vascular interface Annu Rev Biomed Eng 2014 16 16 505 532 24905872 10.1146/annurev-bioeng-071813-104908 PMC4450720

[27] Todd M Lipoedema: presentation and management Br J Community Nurs 2010 15 04 S10 S16 10.12968/bjcn.2010.15.Sup3.47363 20559170

[28] Dadras M Mallinger P J Corterier C C Theodosiadi S Ghods M Liposuction in the treatment of lipedema: a longitudinal study Arch Plast Surg 2017 44 04 324 331 28728329 10.5999/aps.2017.44.4.324 PMC5533060

[29] Tuğral A Bakar Y An approach to lipedema: a literature review of current knowledge of an underestimated health problem Eur J Plast Surg 2019 42 549 558

[30] Wollina U Lipedema-an update Dermatol Ther 2019 32 02 e12805 30565362 10.1111/dth.12805

[31] Poojari A Dev K Rabiee A Lipedema: insights into morphology, pathophysiology, and challenges Biomedicines 2022 10 12 3081 36551837 10.3390/biomedicines10123081 PMC9775665

[32] Warren Peled A Kappos E A Lipedema: diagnostic and management challenges Int J Womens Health 2016 8 389 395 27570465 10.2147/IJWH.S106227 PMC4986968

[33] Vyas A Ghufran A Continuing Education ActivityAvailable at:https://www.ncbi.nlm.nih.gov/books/nbk573066/?report=printable(Accessed on December 19, 2023)

[34] Forner-Cordero I Forner-Cordero A Szolnoky G Update in the management of lipedema Int Angiol 2021 40 04 345 357 33870676 10.23736/S0392-9590.21.04604-6

[35] Esmer M Schingale F J Unal D Yazıcı M V Güzel N A Physiotherapy and rehabilitation applications in lipedema management: a literature review Lymphology 2020 53 02 88 95 33190432

[36] Peprah K MacDougall D Liposuction for the Treatment of Lipedema: A Review of Clinical Effectiveness and GuidelinesLiposuction for the Treatment of Lipedema: A Review of Clinical Effectiveness and Guidelines. Accessed June 7, 2019 at:https://www.ncbi.nlm.nih.gov/books/nbk545818/31479212

[37] Tran K Horton J Liposuction for Lipedema: 2022 Update: Rapid Review Ottawa (ON) Canadian Agency for Drugs and Technologies in Health 2022Aug. Available at:https://www.ncbi.nlm.nih.gov/books/NBK603381/38713790

[38] Duhon B H Phan T T Taylor S L Crescenzi R L Rutkowski J M Current mechanistic understandings of lymphedema and lipedema: tales of fluid, fat, and fibrosis Int J Mol Sci 2022 23 12 6621 35743063 10.3390/ijms23126621 PMC9223758

[39] Alwardat N Di Renzo L Alwardat M The effect of lipedema on health-related quality of life and psychological status: a narrative review of the literature Eat Weight Disord 2020 25 04 851 856 31062201 10.1007/s40519-019-00703-x

[40] Aksoy H Karadag A S Wollina U Cause and management of lipedema-associated pain Dermatol Ther 2021 34 01 e14364 33001552 10.1111/dth.14364

[41] Clarke C Kirby J N Smidt T Best T Stages of lipoedema: experiences of physical and mental health and health care Qual Life Res 2023 32 01 127 137 35972618 10.1007/s11136-022-03216-w PMC9829602

[42] Cannataro R Michelini S Ricolfi L Management of lipedema with ketogenic diet: 22-month follow-up Life (Basel) 2021 11 12 1402 34947933 10.3390/life11121402 PMC8707844

[43] Schlosshauer T Heiss C von Hollen A K Spennato S Rieger U M Liposuction treatment improves disease-specific quality of life in lipoedema patients Int Wound J 2021 18 06 923 931 33955179 10.1111/iwj.13608 PMC8613387

[44] Bouillon V N Hinson C S Hu M Brooks R M Management of lipedema beyond liposuction: a case study Aesthet Surg J Open Forum 2023 5 ojad088 37811191 10.1093/asjof/ojad088 PMC10559941

[45] Podda M Kovacs M Hellmich M A randomised controlled multicentre investigator-blinded clinical trial comparing efficacy and safety of surgery versus complex physical decongestive therapy for lipedema (LIPLEG) Trials 2021 22 01 758 34717741 10.1186/s13063-021-05727-2 PMC8557553

[46] Amato A CM Amato F CM Amato J LS Benitti D A Lipedema prevalence and risk factors in Brazil J Vasc Bras 2022 21 e20210198 35677743 10.1590/1677-5449.202101981 PMC9136687

[47] Czerwińska M Teodorczyk J Hansdorfer-Korzon R A scoping review of available tools in measurement of the effectiveness of conservative treatment in lipoedema Int J Environ Res Public Health 2022 19 12 7124 35742373 10.3390/ijerph19127124 PMC9222339

[48] Falck J Rolander B Nygårdh A Jonasson L L Mårtensson J Women with lipoedema: a national survey on their health, health-related quality of life, and sense of coherence BMC Womens Health 2022 22 01 457 36401222 10.1186/s12905-022-02022-3 PMC9673372

[49] Melander C Juuso P Olsson M Women's experiences of living with lipedema Health Care Women Int 2022 43(1–3):54 69 34252343 10.1080/07399332.2021.1932894

[50] Amato A CM Benitti D A Lipedema can be treated non-surgically: a report of 5 cases Am J Case Rep 2021 22 01 e934406 34871293 10.12659/AJCR.934406 PMC8667633

[51] Bertlich M Jakob M Bertlich I Schift R Bertlich R Lipedema in a male patient: report of a rare case - management and review of the literature GMS Interdiscip Plast Reconstr Surg DGPW 2021 10 Doc11 34660173 10.3205/iprs000161 PMC8495372

[52] Yamamoto T Yamashita M Furuya M Hayashi A Lymph preserving lipectomy under indocyanine green lymphography navigation J Plast Reconstr Aesthet Surg 2015 68 01 136 137 10.1016/j.bjps.2014.08.014 25172437

[53] van de Pas C B Boonen R SM Stevens S Willemsen S Valkema R Neumann M Does tumescent liposuction damage the lymph vessels in lipoedema patients? Phlebology 2020 35 04 231 236 31674863 10.1177/0268355519885217 PMC7178148

[54] Romeijn J RM de Rooij M JM Janssen L Martens H Exploration of patient characteristics and quality of life in patients with lipoedema using a survey Dermatol Ther (Heidelb) 2018 8 02 303 311 29748843 10.1007/s13555-018-0241-6 PMC6002318

[55] Langendoen S I Habbema L Nijsten T EC Neumann H AM Lipoedema: from clinical presentation to therapy. a review of the literature Br J Dermatol 2009 161 05 980 986 19785610 10.1111/j.1365-2133.2009.09413.x

[56] Godoy MdeF Buzato E Brigidio P AF Pereira de Godoy J M Is lymphostasis an aggravant of lipedema? Case Rep Dermatol 2012 4 03 222 226 23185156 10.1159/000342073 PMC3506057

[57] Suga H Araki J Aoi N Kato H Higashino T Yoshimura K Adipose tissue remodeling in lipedema: adipocyte death and concurrent regeneration J Cutan Pathol 2009 36 12 1293 1298 19281484 10.1111/j.1600-0560.2009.01256.x

[58] Kim J H Kim J H Lee Y M Ahn E M Kim K W Yu Y S Decursin inhibits retinal neovascularization via suppression of VEGFR-2 activation Mol Vis 2009 15 1868 1875 19756180 PMC2743803

[59] Siems W Grune T Voss P Brenke R Anti-fibrosclerotic effects of shock wave therapy in lipedema and cellulite Biofactors 2005 24(1-4):275 282 16403988 10.1002/biof.5520240132

[60] GeneOb Project Paolacci S Precone V Acquaviva F Genetics of lipedema: new perspectives on genetic research and molecular diagnoses Eur Rev Med Pharmacol Sci 2019 23 13 5581 5594 31298310 10.26355/eurrev_201907_18292

[61] Bano G Mansour S Brice G Pit-1 mutation and lipoedema in a family Exp Clin Endocrinol Diabetes 2010 118 06 377 380 19609847 10.1055/s-0029-1224154

[62] Precone V Barati S Paolacci S Genetic syndromes with localized subcutaneous fat tissue accumulation Acta Biomedica 2019 90 10 90 92 31577262 10.23750/abm.v90i10-S.8767 PMC7233643

[63] Zechner U Kohlschmidt N Kempf O Familial Sotos syndrome caused by a novel missense mutation, C2175S, in NSD1 and associated with normal intelligence, insulin dependent diabetes, bronchial asthma, and lipedema Eur J Med Genet 2009 52 05 306 310 19545651 10.1016/j.ejmg.2009.06.001

[64] Waxler J L Guardino C Feinn R S Lee H Pober B R Stanley T L Altered body composition, lipedema, and decreased bone density in individuals with Williams syndrome: a preliminary report Eur J Med Genet 2017 60 05 250 256 28254647 10.1016/j.ejmg.2017.02.007 PMC5490490

[65] Zhang Y Nadeau M Faucher F Progesterone metabolism in adipose cells Mol Cell Endocrinol 2009 298(1-2):76 83 18984031 10.1016/j.mce.2008.09.034

[66] Halk A B Damstra R J First Dutch guidelines on lipedema using the international classification of functioning, disability and health Phlebology 2017 32 03 152 159 27075680 10.1177/0268355516639421

[67] Schmeller W Meier-Vollrath I Lipödem - Aktuelles zu einem weitgehend unbekannten krankheitsbild Aktuelle Derm 2007 33 07 251 260

[68] Lontok E T Lipedema: A Giving Smarter GuideAvailable at:https://www.researchgate.net/publication/314134271(Accessed on December 19, 2023)

[69] Book Review. Lipedema - The Disease They Call Fat - Lymphoedema Education Solutions. Accessed Feb 23, 2024 at:https://lymphoedemaeducation.com.au/2017/11/book-review-lipedema-disease-call-fat/

[70] Wounds U K Best Practice Guidelines: The Management of Lipoedema London Wounds UK. Accessed 2017 at:https://www.google.com/search?q=wounds+uk.+best+practice+guidelines%3a+the+management+of+lipoedema.+london%3a+wounds+uk%3b+2017.&rlz=1c5chfa_ensa941sa941&oq=wounds+uk.+best+practice+guidelines%3a+the+management+of+lipoedema.+london%3a+wounds+uk%3b+2017.&gs_lcrp=egzjahjvbwuybggaeeuyodibbzyxngowajsoagcwaga&sourceid=chrome&ie=utf-8

[71] Bertsch T Erbacher G Lipoedema - myths and facts part 1 Phlebologie 2018 47 02 84 92

[72] Jørgensen M G Toyserkani N M Hansen F CG Thomsen J B Sørensen J A Prospective validation of indocyanine green lymphangiography staging of breast cancer-related lymphedema Cancers (Basel) 2021 13 07 1540 33810570 10.3390/cancers13071540 PMC8063087

[73] Fetzer A Wise C Living with lipoedema: reviewing different self-management techniques Br J Community Nurs 2015 Suppl Chronic S14 S19, S16–S19 26418584 10.12968/bjcn.2015.20.Sup10.S14

[74] Kinavey H Cool C The broken lens: how anti-fat bias in psychotherapy is harming our clients and what to do about it Women Ther 2019 42(1–2):116 130

[75] Fetzer A Women in dire need: the far-reaching impact of lipoedema on women's lives Br J Community Nurs 2020 25(Sup4):S6 S9 10.12968/bjcn.2020.25.Sup4.S6 32271101

[76] Dudek J E Białaszek W Gabriel M Quality of life, its factors, and sociodemographic characteristics of Polish women with lipedema BMC Womens Health 2021 21 01 27 33446179 10.1186/s12905-021-01174-y PMC7809838

[77] Erbacher G Bertsch T Lipoedema and pain: what is the role of the psyche? Results of a pilot study with 150 patients with lipoedema Phlebologie 2020 49 05 305 316

[78] Szolnoky G Borsos B Bársony K Balogh M Kemény L Complete decongestive physiotherapy with and without pneumatic compression for treatment of lipedema: a pilot study Lymphology 2008 41 01 40 44 18581957

[79] Fetzer A Specialist approaches to managing lipoedema Br J Community Nurs 2016 21(suppl):S30 S35 10.12968/bjcn.2016.21.Sup4.S30 27046426

[80] Shin B W Sim Y J Jeong H J Kim G C Lipedema, a rare disease Ann Rehabil Med 2011 35 06 922 927 22506222 10.5535/arm.2011.35.6.922 PMC3309375

[81] Lohrmann C Foeldi E Langer M MR imaging of the lymphatic system in patients with lipedema and lipo-lymphedema Microvasc Res 2009 77 03 335 339 19323976 10.1016/j.mvr.2009.01.005

[82] Reich-Schupke S Altmeyer P Stücker M Thick legs - not always lipedema J Dtsch Dermatol Ges 2013 11 03 225 233 23231593 10.1111/ddg.12024

[83] International Union of Phlebology Lee B B Andrade M Antignani P L Diagnosis and treatment of primary lymphedema. Consensus document of the International Union of Phlebology (IUP)-2013 Int Angiol 2013 32 06 541 574 24212289

[84] Rudkin G H Miller T A Lipedema: A clinical entity distinct from lymphedema Plast Reconstr Surg 1994 94 06 841 849 7972431

[85] Wright T F Herbst K L A case series of lymphatic injuries after suction lipectomy in women with lipedema Am J Case Rep 2022 23 e935016 35811389 10.12659/AJCR.935016 PMC9284075

[86] Baumgartner A Hueppe M Meier-Vollrath I Schmeller W Improvements in patients with lipedema 4, 8 and 12 years after liposuction Phlebology 2021 36 02 152 159 32847472 10.1177/0268355520949775

[87] Sandhofer M Hanke C W Habbema L Prevention of progression of lipedema with liposuction using tumescent local anesthesia: results of an international consensus conference Dermatol Surg 2020 46 02 220 228 31356433 10.1097/DSS.0000000000002019

[88] Mortada H Alaqil S Jabbar I A Safety and effectiveness of liposuction modalities in managing lipedema: systematic review and meta-analysis Arch Plast Surg 2024 51 05 510 526 39345998 10.1055/a-2334-9260 PMC11436335

[89] Campisi C C Ryan M Boccardo F Campisi C Fibro-lipo-lymph-aspiration with a lymph vessel sparing procedure to treat advanced lymphedema after multiple lymphatic-venous anastomoses: The complete treatment protocol Ann Plast Surg 2017 78 02 184 190 27404468 10.1097/SAP.0000000000000853

[90] Campisi C C Ryan M Boccardo F Campisi C Prevention of lymphatic injuries with lymphatic mapping: the combined technique J Plast Reconstr Aesthet Surg 2016 69 08 e164 e165 27245146 10.1016/j.bjps.2016.04.025

[91] Pfister C Dawczynski H Schingale F J Selenium deficiency in lymphedema and lipedema—a retrospective cross-sectional study from a specialized clinic Nutrients 2020 12 05 1211 32344864 10.3390/nu12051211 PMC7281982

[92] Hodge L M King H H Williams A G Jr Abdominal lymphatic pump treatment increases leukocyte count and flux in thoracic duct lymph Lymphat Res Biol 2007 5 02 127 133 17935480 10.1089/lrb.2007.1001

[93] Schaverien M V Moeller J A Cleveland S D Nonoperative treatment of lymphedema Semin Plast Surg 2018 32 01 17 21 29636649 10.1055/s-0038-1635119 PMC5891656

[94] Bellini E Grieco M P Raposio E A journey through liposuction and liposculture: review Ann Med Surg (Lond) 2017 24 53 60 29158895 10.1016/j.amsu.2017.10.024 PMC5681335

